# Facing Anxiety, Growing Up. Trait Emotional Intelligence as a Mediator of the Relationship Between Self-Esteem and University Anxiety

**DOI:** 10.3389/fpsyg.2019.00567

**Published:** 2019-03-15

**Authors:** Rocio Guil, Rocio Gómez-Molinero, Ana Merchan-Clavellino, Paloma Gil-Olarte, Antonio Zayas

**Affiliations:** ^1^Instituto de Desarrollo Social y Sostenible (INDESS), University of Cádiz, Cádiz, Spain; ^2^Psychology Department, University of Cádiz, Cádiz, Spain

**Keywords:** trait emotional intelligence, state anxiety, trait anxiety, self-esteem, emotion regulation

## Abstract

The current study analyzed how trait emotional intelligence (trait EI) mediates the relationship between self-esteem and state anxiety and trait anxiety. The sample was composed of 153 undergraduate students from the University of Cádiz, Spain (71.9% women and 28.1% men). Students completed measures of self-esteem, state anxiety, trait anxiety, and trait EI. Mediation analyses were completed with three trait EI dimensions (EA, emotional attention; EC, emotional clarity; and MR, mood repair) as mediating variables, self-esteem as the independent variable, and state anxiety and trait anxiety as the dependent ones. Our results confirmed that self-esteem scores explained and predicted both, state and trait anxiety values (13% for state and 21% for trait anxiety). This explanatory capacity is increased by 8% when accounting for all trait EI dimensions. Considering state anxiety, the results of the direct effects showed that a decrease in their levels is predicted through the increases in the levels of both, self-esteem and MR. Regarding trait anxiety, the results of the direct effects showed that a decrease in their levels is predicted, in addition to an increment of self-esteem and MR values, by an increase of EC and a decrease of EA. Conversely, indirect effects revealed that higher levels of self-esteem were associated with worse scores in EA and worse MR, which in turn would enhance both state and trait anxiety levels. Moreover, regarding trait anxiety higher levels of self-esteem were associated with worse scores in EA and worse EC, therefore increasing trait anxiety levels. As shown, the negative association found between self-esteem and EA becomes a key element. The effect of self-esteem on EA and the influence that the latter had on EC and MR exerts an indirect mediated effect with the power to invert the influence that self-esteem wields on both types of anxiety. In this sense, the apparent protective role of self-esteem changed, turning into a risk factor that promotes higher anxiety values.

## Introduction

University stage appears as a period of significant change, in which the transition from adolescence to adulthood, academic pressures, and different social and personal responsibilities arise. Besides, new evaluation systems and fear of failure can awake negative feelings that may alter academic performance. Hence, the efforts that students make to acclimate to this new context can cause undesirable side effects. In this sense, and according to the World Health Organization [WHO], 2017, in Spain, 2.408.700 people were affected by anxiety disorders in 2017. Precisely, this report pointed out university students as a risk group due to the academic, personal, psychological, social, and strain levels they have to face.

Anxiety is an emotional reaction that appears when people face unknown, aversive or anticipated events and is aggravated when a person experiences extended, unresolved or multiple stressors (Lazarus and Folkman, 1984). However, while mild anxiety may have a positive influence on academic outcomes, increasing efficiency, and intellectual functioning, high levels are detrimental to academic performance and may result in maladaptive behaviors. According to Spielberger (1966, 1972,1989), to adequately conceptualize this construct, it is crucial to discriminate between anxiety as an emotional state and anxiety as a personality trait. As the State-Trait Anxiety Theory posits (Cattell and Scheier, 1961), state anxiety refers to an immediate emotional state, transient and modifiable in time that causes tension and activation of the autonomic nervous system. For example, the fact of facing an exam as a specific situation can raise state anxiety levels, caused by the fear of failure before that event occurs. In turn, trait anxiety is defined as a propensity to be anxious. It is a stable personality trait, determined by an inclination to perceive (or not) stimuli as threatening (Spielberger, 1972, 1980; Spielberger and Díaz-Guerrero, 1975). Students with high levels of trait anxiety perceive a broader range of situations as hostile and are more predisposed to suffer from state anxiety with a higher frequency and intensity.

The research focused on the incidence and the effect of anxiety on psychological adjustment to the university environment has shown diverse results. This diversity is probably due to the different measurement instruments used that differ in the way of approaching this construct. In this sense, while some authors have focused on the negative influence of test anxiety, characterized as occasional anxiety and similar to the state anxiety construct developed by Cattell and Scheier (1961), others based their research on a stable disposition to be anxious, comparable to trait anxiety. However, we rarely found studies that explicitly distinguish between these two types of anxiety. Therefore, it is crucial to discriminate between these two constructs to determine which psychosocial variables act as protection and (or) risk factors to state and trait anxiety. This would provide empirical support to design distinct interventions aimed to decrease students’ anxiety when facing both, general and specific situations.

In this sense, self-esteem is one of the constructs more often linked to anxiety (Sowislo and Orth, 2013). One of the most accepted definitions is Rosenberg (1965), who describes self-esteem as the feeling of satisfaction and the confidence that people have in themselves (Pearlin, 1989). Besides, and according to the Terror Management Theory (Greenberg et al., 1986), self-esteem can act as a protective factor against anxiety, given that the perception that one is good (self-esteem) has the defensive goal of minimizing anxiety, serving, thus, as a control mechanism against terror. More recently, Manna et al. (2016) explored the relationship between self-esteem, anxiety, and depression, and evaluated two main models: *the scar model*, where depression and anxiety can be a consequence of low self-esteem; and *the vulnerability model*, in which low self-esteem predicted depression and anxiety. The findings suggested that the effects of self-esteem on depression and anxiety were significantly higher than the effects of anxiety and depression on self-esteem.

The empirical evidence seems to confirm both, Terror Management Theory and vulnerability model (Sowislo and Orth, 2013; Liu et al., 2014; Bajaj et al., 2016) stressing that high self-esteem is a protective factor against mental health problems in young adults and adolescents (Wilburn and Smith, 2005; Sharaf et al., 2009). In addition, the literature also highlights the importance of self-esteem when it comes to manifest both trait and state anxiety (Cardenal Hernáez and Díaz Morales, 2000; Garaigordobil et al., 2003; Núñez et al., 2006). In this sense, Fathi-Ashtiani et al. (2007) pointed out that an adequate self-concept and an inclination to high levels of self-esteem not only protected from state but from trait anxiety. In a Spanish sample, Cabanach et al. (2015) noted that when the student’s self-assessment is low, the risk of perceiving factors in the academic context as threats increased. In addition, and more recently, Jirdehi et al. (2018) pointed out that low levels of self-esteem might have multiple negative consequences. Following these authors, a person with low self-esteem shows difficulties expressing their interests and rights what in turn leads to feelings of anguish, frustration, and stress, unlike those who displayed higher self-esteem levels that show greater success in their actions.

Likewise, research on emotion has demonstrated the importance of emotion regulation in the adaptation process and well-being (e.g., Gil-Olarte et al., 2009; Peña-Sarrionandia et al., 2015; Mayer et al., 2016; Mestre et al., 2017). In fact, emotional dysregulation can increase anxiety, produce a lack of social competence, and may undermine decision-making processes (Loewenstein and Lerner, 2003; Wills et al., 2016; Hartman et al., 2017). In this sense, trait emotional intelligence (trait EI) appears as an important protective factor against anxiety among university students. Trait EI refers to the self-perception of our emotional abilities, that is, how good we think we are in terms of understanding, regulating, and expressing our emotions responsible for maintaining an adequate level of well-being (Petrides et al., 2016). More specifically, trait EI describes the self-perceptions of our emotional world (Petrides et al., 2018). Besides, Bandura and his colleagues found that beliefs in owns emotional abilities (self-efficacy) was related, prospectively, with less depressive symptoms and more pro-social behaviors (Bandura et al., 2003; Caprara et al., 2008). Similarly, Tamir et al. (2007) found that la self-efficacy perceptions in emotion regulation, measured early in the first year among college students, was associated with less negative emotions, more positive emotions, greater psychological well-being, and lower levels of depression at the end of the year (see also Goldin et al., 2012). Recently, Bigman et al. (2016) demonstrated that positive expectations in emotion regulation are equally powerful modifying the way people regulate their emotions and their feelings. Hence, beliefs about success in emotion regulation are positively associated with desirable emotional outcomes. One of the most widely used instruments to evaluate trait EI is the Trait Meta-Mood Scale (TMMS, Salovey et al., 1995). TMMS is a self-reported measure, composed of three subscales: *emotional attention* (EA) that refers to the self-perceptions regarding the degree to which an individual attends to his or her emotional experiences, *emotional clarity* (EC) that refers to self-perceptions concerning how clearly people understand own and other’s emotional states, and *mood repair* (MR) that represents the self-perceptions regarding the ability to adequately manage emotions. Therefore, this instrument assesses the perception that a person has about their own ability to attend, understand and modify their emotional states.

Research concerning trait EI (assessed with TMMS) and anxiety is abundant, although we have found different empirical results depending on the trait EI dimension evaluated. With regard to EA, literature does not show consensus on the role played by this construct in anxiety disorders. In this sense, we found studies that suggested a negative relationship between EA and social anxiety. According to Turk et al. (2005), socially anxious students reported a greater difficulty in dealing with emotions in a sample of 766 undergraduate students. There are also studies that indicate the potentially detrimental effect of EA, relating it with greater affective instability and emotional variability (Thompson et al., 2009; Thompson, 2011). Likewise, in a Spanish university sample, Pena and Losada (2017) suggested that high EA is not necessarily beneficial, showing a positive relationship between EA and state anxiety. However, Ghorbani et al. (2002) and Salovey et al. (2002) found no relationship between social anxiety and EA. Regarding the above mention, Butler et al. (2006) emphasized the need to further investigate this relationship in order to determine whether greater EA is linked (or not) to positive outcomes in the context of anxiety disorders.

Concerning EC research has highlighted its importance on emotion regulation, since the ability to clearly identify one’s emotions is the first step for a successful emotion regulation and coping (Butler et al., 2006). In this case, most of the studies agree to establish a negative relationship between this construct and anxiety levels. Specifically, lower levels of EC have been related to worse psychosocial adjustment (e.g., Salguero et al., 2012) and higher levels of anxiety (Ghorbani et al., 2002). On the contrary, greater EC has been linked to positive coping, greater well-being (Gohm and Clore, 2002), and less anxiety (Salovey et al., 2002; O’Toole et al., 2013; Thompson et al., 2017).

Finally, regarding the belief in one’s ability to regulate emotional states, the literature points out positive correlations between expectations in successful emotion regulation and emotional results (Bigman et al., 2016) finding a negative relationship between MR and anxiety (Ghorbani et al., 2002). Specifically, in the university context, it has been found that students who expected to be more successful in regulating their negative emotions displayed lower test anxiety, had better test performance, and presented fewer depressive symptoms (Catanzaro and Mearns, 1990). Altogether, these results seem to confirm a well-establish relationship between self-esteem and trait EI (Cheung et al., 2015), supporting the hypothesis that self-perceptions regarding emotional abilities are an essential contributor to psychological adjustment (Fernández-Berrocal et al., 2006; Petrides et al., 2016).

Hence, while it is well known the direct relationships between the self-esteem, trait EI, and anxiety, so far there is no empirical evidence about the mechanism through which trait EI (EA, EC, and MR) mediates the relationship between self-esteem and state and trait anxiety. Hence, the goal of the present study is to develop and test a model that considered EA, EC, and MR as mediator variables that enhance the effects of self-esteem on state and trait anxiety in university students.

## Materials and Methods

### Participants and Procedure

Participants were 153 undergraduate students divided into fist cycle of studies (first and second year) and second cycle of studies (third and fourth study years). Students were selected by quota sampling from Social Sciences Degree Program of the University of Cádiz, Spain. Precisely, students were attending to Psychology and Human Resources Degree. The majority of students were women (71.9%) and the average age was 21.38 (range = 18–48, *SD* = 3.76). Participants were given a paper-and-pencil questionnaire with all the scales presented at the same time. Students had no time limitation and the questionnaires were completed during their leisure time. Participation was anonymous and voluntary. Data were collected following the general principles and the ethical research standards of the American Psychological Association (APA). An ethics approval was not required as per the University of Cádiz’s guidelines and national regulations. All subjects gave written informed consent in accordance with the Declaration of Helsinki. They received no financial compensation for participation in the study.

### Instruments

#### Trait and State Anxiety

Trait and state anxiety were assessed with the Spanish adaptation of the State-Trait Anxiety Inventory (STAI, Spielberger et al., 2011). The STAI has 40 items, 20 items allocated to each of the two independent anxiety scales: state and trait anxiety. Responses for the state anxiety scale assesses the intensity of current feelings “at this moment” on a 4-point Likert scale ranging from 0 (*not at all*) to 3 (*very much so*). Responses for the trait anxiety scale measures the frequency of feelings “in general” on a 4-point Likert scale ranging from 0 (*almost never*) to 3 (*almost always*). State anxiety example items were “I am tense; I am worried” and “I feel calm; I feel secure.” Trait anxiety items include “I worry too much over something that really doesn’t matter” and “I am content; I am a steady person.” Higher scores indicate greater anxiety levels. The psychometric properties of this instrument have been provided, in general, good results, both in the original version and in the Spanish adaptation with Cronbach alphas above 0.80. In the current sample, reliability coefficients were 0.89 for trait anxiety and 0.92 for state anxiety.

#### Self-Esteem

Self-esteem was assessed with the Spanish adaptation of the Rosenberg’s Self-esteem Scale (RSES, Rosenberg, 1965). This instrument is composed of 10 items that measure global self-worth by measuring both, positive and negative feelings about the self. The RSES contains an equal number of positively (e.g., people feeling satisfied with life) and negatively (e.g., people feeling they are failures) worded items. The scale is believed to be unidimensional. Responses were given on a 4-point Likert scale ranging from 1 (*strongly agree*) to 4 (*strongly disagree*). Example items were “I feel that I am a person of worth, at least on an equal plane with others” and “I certainly feel useless at times.” Higher scores indicate greater self-esteem levels. Several studies have shown their psychometric properties, supporting their use in different languages (Rosenberg, 1965; Silber and Tippett, 1965; Kaplan and Pokorny, 1969; Kernis et al., 1991; Hagborg, 1993; Vázquez Morejón et al., 2004). Cronbach alpha in the present studio was 0.88.

#### Trait Emotional Intelligence

Trait Emotional Intelligence was assessed with the Spanish version of Trait-Meta Mood Scale (TMMS-24, Fernández-Berrocal et al., 1998). The original scale was developed by Salovey et al. (1995). This is a self-report instrument and evaluates the extent to which people reflect upon their moods. TMMS-24 comprised 24 items that include three key dimensions of emotional intelligence: EA (eight items), EC (eight items), and MR (eight items). EA refers to the ability to attend and to express moods and emotions, EC is the ability to understand and differentiate moods and emotions, and MR denotes the ability to maintain positive emotions in order to repair negative ones (Salovey et al., 1995). Responses were given on a 5-point Likert scale ranging from 1 (*completely disagree*) to 5 (*totally agree*). Example items were “I often think about my feelings” and “I pay a lot of attention to how I feel.” Higher scores indicate greater trait EI levels. The Spanish adaptation showed an internal consistency, for all subscales, above 0.85. Cronbach alphas for each of the subscales in the present studio were 0.90 for EA, 0.90 for EC, and 0.85 for MR.

### Statistical Analysis

Preliminary analyses were carried out to compute descriptive statistics, internal consistencies, bivariate correlations, and hierarchical regression analyses using SPSS software (version 20.0; IBM, Chicago, IL, United States). To determine whether EA, EC, and MR mediated the relationship between the self-esteem and state and trait anxiety, serial mediation analyses were performed using Model 6 in the PROCESS tool (Hayes, 2018). PROCESS is an SPSS macro for mediation, moderation, and conditional process modeling that allows for one independent variable, one dependent variable, and more than one simultaneous mediator variables. To verify which indirect effect had more statistical weight, we performed specific contrasts for indirect effects. As a statistical significance criterion, we used the 95% confidence interval (CI) generated by the bias-corrected bootstrap method set to 10.000 reiterations.

## Results

### Descriptive Statistics and Analysis of Variance

Table 1 displays reliability coefficients, descriptive statistics for the total sample and correlations among all variables involved in the study. State and trait anxiety average levels are under the theoretical mean, while all other variables are above it. Internal consistencies of all scales administered were above 0.85. As expected, state and trait anxiety were significantly and negatively correlated with self-esteem. Moreover, both anxiety types were negatively correlated with EC and MR while trait anxiety was positively associated with EA. Finally, age was significantly and positively correlated with self-esteem.

**Table 1:** Descriptive statistics for total sample, Cronbach’s α values, and correlations among all study variables.

	M	SD	α	Correlations								
	
Variables				1	2	3	4	5	6	7	8	9	1
State	0.82	0.51		1	–	–	–	–	–	–	–	–	–
Trait	1.01	0.49		0.62**	1	–	–	–	–	–	–	–	–
Age	21.72	3.76		–0.00	–0.11	1	–	–	–	–	–	–	–
Sex				0.02	–0.15	0.15	1	–	–	–	–	–	–
Year				0.06	–0.14	0.31**	0.37**	1	–	–	–	–	–
Degree				0.13	–0.03	0.28**	0.26**	0.26**	1	–	–	–	–
SFE	3.17	0.59	0.88	–0.36**	–0.67**	0.21*	0.10	0.23**	0.23**	1	–	–	–
EA	3.41	0.82	0.90	0.02	0.26**	–0.10	–0.04	–0.21*	–0.20*	–0.17**	1	–	–
EC	3.40	0.77	0.90	–0.27**	–0.38**	0.12	0.11	0.18	–0.41	0.37**	0.09	1	–
MR	3.47	0.75	0.85	–0.42**	–0.45**	0.09	–0.01	–0.01	–0.04	0.48**	0.17**	0.46**	1


*^∗∗^p < 0.01 (2-tailed); ^∗^*p* < 0.05 (2-tailed); State, state anxiety; Trait, trait anxiety; Year, cycle of studies; Degree, academic degree; SFE, self-esteem; EA, emotional attention; EC, emotional clarity; MR, mood repair.*

Two hierarchical regressions were conducted for each outcome variable to check whether EA, EC, MR, and self-esteem predicted state and trait anxiety after controlling for the influence of age, gender, degree, and year of studies (first and second cycle). At step 1, we entered age, gender, degree, and year of studies as control variables, and at step 2 EA, EC, MR, and self-esteem as predictor variables. The results of the hierarchical regression 1 (outcome variable state anxiety) indicated that the four predictors explained 21.1% of the variance (*R*^2^ = 0.21, *F*[8,142] = 5.31, *p* < 0.01). It was found that self-esteem significantly predicted state anxiety (β = -0.24, *p* < 0.01), as did MR (β = -0.29, *p* < 0.01). Gender, age, academic domain, and year of studies were not associated with state anxiety. The results of the hierarchical regression 2 (outcome variable trait anxiety) indicated that the four predictors explained 50.4% of the variance (*R*^2^ = 0.50, *F*[8,142] = 21.91, *p* < 0.01). It was found that self-esteem significantly predicted trait anxiety (β = -0.53, *p* < 0.01), as did EA (β = 0.24, *p* < 0.01), and MR (β = -0.19, *p* < 0.01). Gender, age, academic domain, and year of studies were not associated with trait anxiety.

### Mediation Analysis

To further analyze the relationship between all variables studied, mediation analyses were conducted to test whether EA, EC, and MR mediated the relationship between self-esteem and state and trait anxiety. Specifically, two serial mediation models were proposed. Model A included EA, EC, and MR as mediator variables, self-esteem as the independent variable, and state anxiety as the dependent one. In turn, Model B included the same mediators and independent variable but trait anxiety as the dependent one. In serial mediation, mediators have a direct effect on each other and it is assumed that the independent variable (self-esteem) affects the mediators in a serial manner (Hayes, 2013), lastly influencing the dependent variables.

Regarding model A (Table 2), the total amount of variance explained by the overall model was 21% (*R*^2^ = 0.21). Specifically, the analysis revealed that self-esteem accounted for 13% of the unique variance of state anxiety (*R*^2^ = 0.13; c: *B* = -0.31, *p* = 0.000). Considering the direct effects, self-esteem (c′: β = -0.16, *p* = 0.03) and MR (b_3_: β = -0.20, *p* = 0.001) were negatively related to state anxiety, suggesting that students with higher levels of self-esteem and higher levels of MR showed low levels of trait anxiety. Neither attention nor clarity showed statistically significant direct effects. Other statistically significant direct effects linked self-esteem with a decrease in EA (a_1_: β = -0.24, *p* = 0.032) and an increase in EC (a_2_: β = 0.51, *p* = 0.000) and MR (a_3_: β = 0.51, *p* = 0.000). Moreover, EA was positively related to EC (d_21_: β = 0.14, *p* = 0.042) and MR (d_31_: β = 0.19, *p* = 002), and EC to MR (d_32_: β = 0.29, *p* = 0.000).

**Table 2:** Serial Mediator Model A: Model summary, total effect, direct effect, indirect effect, and main specific indirect effect contrast definitions.

**Model Summary**	***R*^2^**	**MSE**	***F***	***df*1**	***df*2**	***p* (sig.)**	
	
Total effect model	0.2126	0.21	9.99	4.00	148.00	0.000	
SFE on state	0.1317	0.2270	22.89	1.00	151.00	0.000	
						**95% CI**	
	**Path**	**Coefficient**	**BootSE**	**T**	**P**	**BootLL**	**BootUL**
	
Total effect of SFE on state, without accounting for EA, EC and MR	C	–0.3151	0.06	–4.78	0.000	–0.44	–0.18
Total effect of SFE on state, when accounting for EA, EC and MR	c′	–0.1638	0.07	–2.13	0.034	–0.31	–0.01
Direct effect of SFE on EA	*a*_1_	–0.2415	0.11	–2.15	0.032	–0.46	–0.02
Direct effect of SFE on EC	*a*_2_	0.5119	0.09	5.15	0.000	0.31	0.70
Direct effect of SFE on MR	*a*_3_	0.5130	0.09	5.60	0.000	0.33	0.69
Direct effect of MR on state	*b*_3_	–0.2084	0.06	–3.33	0.001	–0.33	–0.08
Direct effect of EA on EC	*d*_21_	0.1452	0.07	2.04	0.042	0.00	0.28
Direct effect of EA on MR	*d*_31_	0.1929	0.06	3.15	0.002	0.07	0.31
Direct effect of EC on MR	*d*_32_	0.2915	0.06	4.20	0.000	0.15	0.42
**Indirect effect**							
Ind_ 3_ via EA and MR	*a*_1_*d*_31_*b*_3_	0.0097	0.00			0.00	0.03
Ind_ 4_ via EA, EC, and MR	*a*_1_*d*_21_*d*_32_*b*	0.0021	0.00			0.00	0.00
Ind_6_ via EC and MR	*a*_2_*d*_32_*b*_3_	–0.0311	0.01			–0.07	–0.00
Ind_7_ via MR	*a*_3_*b*_3_	–0.1069	0.04			–0.20	–0.03
**Specific indirect effect contrast definitions**							
Ind_3_ minus Ind_4_	*C12*	0.0076	0.0067			0.00	0.03
Ind_3_ minus Ind_6_	*C14*	0.0408	0.0180			0.01	0.08
Ind_3_ minus Ind_7_	*C15*	0.1166	0.0447			0.04	0.21
Ind_4_ minus Ind_6_	*C17*	0.0332	0.0165			0.01	0.07
Ind_4_ minus Ind_7_	*C18*	0.1091	0.0421			0.03	0.20
Ind_6_ minus Ind_7_	*C21*	0.0758	0.0363			0.01	0.16


*Coefficient non-standardized B coefficients; BootSE, bootstrapping standard errors; CI, bias-corrected and accelerated 95% confidence interval; BootLL, bootstrapping lower limit; BootUL, bootstrapping upper limit. State, state anxiety; SFE, self-esteem; EA, emotional attention; EC, emotional clarity, MR, mood repair.*

**FIGURE 1:**
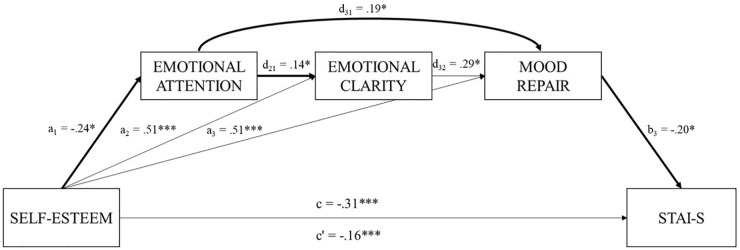
Illustration of the direct and indirect effects model for serial mediation model. In this model EA, EC, and MR mediate the relationship between self-esteem and state anxiety.

Four specific indirect effects presented statistical significance in model A (95% bias-corrected CI). Indirect effect 3 (ind_3_ = *a*_1_*d*_31_*b*_31_), revealed that greater self-esteem was associated with higher levels of state anxiety acting serially through EA and MR (β = 0.0097; *SE* = 0.00; 95% CI = 0.00, 0.03). Indirect effect 4 (ind_4_ = *a*_1_*d*_21_*d*_32_*b*) showed that higher levels of self-esteem also increased state anxiety values but by acting serially through EA, EC, and MR (β = 0.0021; *SE* = 0.00; 95% CI: 0.00, 0.00). Indirect effect 6 (ind_6_ = *a*_2_*d*_32_*b*_3)_ showed that higher levels self-esteem were associated with better scores in EC and better MR, which in turn, decrease state anxiety levels (β = -0.0311; *SE* = 0.01; 95% CI: -0.07, -0.00). Finally, indirect effect 7 (ind_7_ = *a*_3_*b*_3_), revealed that greater levels of self-esteem were associated with higher levels of MR what in turn decreased state anxiety values (β = -0.1069, *SE* = 0.04, 95% CI: -0.20, -0.03). The analysis diagram of model A is shown in Figure 1.

To determine which of the indirect effects had more statistical weight, we performed contrast analyzes between mediators. Table 2 shows the statistically significant contrasts with a CI of 95%. Considering the sign of the coefficients, the analyses showed that the effect of self-esteem on state anxiety acting serially through EA and ME (ind_3_) is the greatest indirect effects. Hence, students with higher levels of self-esteem pay less attention to their emotions what contributed to lower their MR levels hence increasing their state anxiety.

Regarding model B (Table 3), the total amount of variance explained by the overall model, including self-esteem and the mediator variables, was 52% (*R*^2^ = 0.52). Specifically, the analysis revealed that self-esteem accounted for 45% of the unique variance of trait anxiety (*R*^2^ = 0.45; c: *B* = -0.56, *p* = 0.000). Considering the direct effects, self-esteem (c′: β = -0.41, *p* = 0.034), EC (b_2_: β = -0.08, *p* = 0.046), and MR (b_3_: β = -0.12, *p* = 0.009) are negatively related to trait anxiety, suggesting that students with higher levels of self-esteem, higher levels of EC, and higher levels of MR reported lower levels of trait anxiety. On the contrary, higher levels of EA were associated with an increase on trait anxiety (b_1_: β = 0.13, *p* = 0.000). Other statistically significant direct effects showed that self-esteem is negatively related to EA (a_1_: β = -0.24, *p* = 0.032) and positively related to EC (a_2_: β = 0.51, *p* = 0.000) and MR (a_3_: β = 0.51, *p* = 0.000). Moreover, EA was positively associated with EC (d_21_: β = 0.15, *p* = 0.042) and MR (d_31_: β = 0.19, *p* = 0.002), and EC to MR (d_32_: β = 0.29, *p* = 0.000).

**Table 3:** Serial Mediator Model B: Model summary, total effect, direct effect, indirect effect, and main specific indirect effect contrast definitions.

**Model Summary**	***R*^2^**	**MSE**	**F**	**df1**	**df2**	**p (sig.)**	
	
Total effect model	0.5231	0.1182	40.58	4.00	148.00	0.000	
SFE on trait	0.4497	0.1337	123.40	1.00	151.00	0.000	
						**95% CI**	
	**Path**	**Coefficient**	**SE**	**T**	**P**	**BootLL**	**BootUL**
	
Total effect of SFE on trait, without accounting for EA, EC and MR	C	–0.5613	0.0505	–11.10	0.000	–0.66	–0.46
Total effect of SFE on trait, when accounting for EA, EC and MR	c′	–0.4149	0.0576	–7.20	0.000	–0.52	–0.30
Direct effect of SFE on EA	*a*_1_	–0.2415	0.1120 -2.16	0.032	-0.46	–0.02
Direct effect of SFE on EC	*a*_2_	0.5119	0.0993 5.16	0.000	0.32	0.71
Direct effect of SFE on MR	*a*_3_	0.5130	0.0915	5.61	0.000	0.33	0.69
Direct effect of EA on trait	*b*_1_	0.1302	0.0361	3.60	0.000	0.06	0.20
Direct effect of EC on trait	*b*_2_	–0.0844	0.0420	–2.01	0.046	–0.16	–0.00
Direct effect of MR on trait	*b*_3_	–0.1235	0.0469	–2.63	0.009	–0.21	–0.03
Direct effect of EA on EC	*d*_21_	0.1452	0.0700	2.04	0.042	0.00	0.28
Direct effect of EA on MR	*d*_31_	0.1929	0.0600	3.15	0.002	0.07	0.31
Direct effect of EC on MR	*d*_32_	0.2915	0.0600	4.20	0.000	0.15	0.42
**Indirect effect**							
Ind_ 1_ via EA	*a*_1_*b*_1_	–0.0314	0.0210			–0.08	–0.00
Ind_ 2_ via EA and EC	*a*_1_*d*_21_*b_2_*	0.0030	0.0031			0.00	0.01
Ind_ 3_ via EA and MR	*a*_1_*d*_31_*b_3_*	0.0058	0.0045			0.00	0.02
Ind_ 4_ via EA, EC, and MR	*a*_2_*d*_21_*d*_32_*b*_3_	0.0013	0.0013			0.00	0.00
Ind_5_ via EC	*a*_2_*b*_2_	–0.0432	0.0242			–0.10	–0.00
Ind_6_ via EC and MR	*a*_2_*d*_32_*b*_3_	–0.0184	0.0099			–0.04	–0.00
Ind_7_ via MR	*a*_3_*b*_3_	–0.0634	0.0301			–0.13	–0.01
**Specific indirect effect contrast definitions**							
Ind_3_ minus Ind_2_	*C1*	0.0076	0.0067			0.00	0.03
Ind_1_ minus Ind_3_	*C2*	0.0408	0.0180			0.01	0.08
Ind_1_ minus Ind_4_	*C3*	0.1166	0.0447			0.04	0.21
Ind_2_ minus Ind_5_	*C9*	0.0332	0.0165			0.01	0.07
Ind_2_ minus Ind_6_	*C10*	0.1091	0.0421			0.03	0.20
Ind_2_ minus Ind_7_	*C11*	0.0758	0.0363			0.01	0.16
Ind_3_ minus Ind_4_	*C12*	0.0045	0.0042			0.00	0.02
Ind_2_ minus Ind_5_	*C13*	0.0489	0.0241			0.01	0.10
Ind_3_ minus Ind_6_	*C14*	0.0242	0.0120			0.00	0.05
Ind_3_ minus Ind_7_	*C15*	0.0691	0.0323			0.01	0.14
Ind_4_ minus Ind_5_	*C16*	0.0445	0.0243			0.00	0.10
Ind_4_ minus Ind_6_	*C17*	0.0197	0.0107			0.00	0.04
Ind_4_ minus Ind_7_	*C18*	0.0646	0.0305			0.01	0.13
Ind_6_ minus Ind_7_	*C21*	0.0449	0.0253			0.00	0.10


*Coefficient non-standardized B coefficients; BootSE, bootstrapping standard errors; CI, bias-corrected and accelerated 95% confidence interval; BootLL, bootstrapping lower limit; BootUL, bootstrapping upper limit. Trait, trait anxiety; SFE, self-esteem; EA, emotional attention; EC, emotional clarity; MR, mood repair.*

**FIGURE 2:**
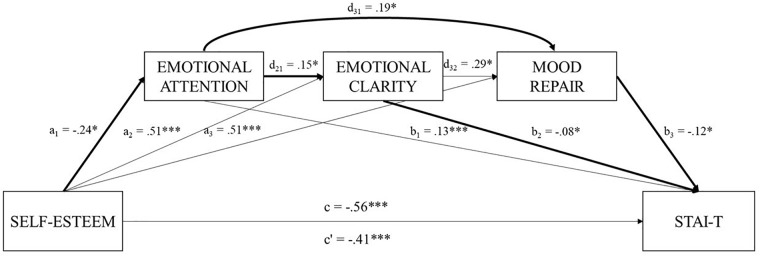
Illustration of the direct and indirect effects model for serial mediation model. In this model EA, EC, and MR mediate the relationship between self-esteem and trait anxiety.

Seven specific indirect effects presented statistical significance in model B (95% bias-corrected CI). Indirect effect 1 (Ind_1_ = *a*_1_*b*_1_), revealed that higher levels self-esteem decrease EA values and, in turn, trait anxiety levels are diminished (β = -0.0314; *SE* = 0.02; 95% CI = -0.08, -0.00). Indirect effect 2 (Ind_2_ = *a*_1_*d*_21_*b_2_*) showed that greater levels of self-esteem increased trait anxiety values by acting serially through EA and EC (β = 0.0030; *SE* = 0.00; 95% CI: 0.00, 0.01). Indirect effect 3 (Ind_3_ = *a*_2_*d*_31_*b*_3)_ indicated that higher levels self-esteem were associated with low scores in EA and low MR, which in turn, increase trait anxiety levels (β = -0.0311; *SE* = 0.01; 95% CI: -0.07, -0.00). Indirect effect 4 (Ind_4_ = *a*_2_*d*_21_*d*_32_*b*_3_) showed that higher levels of self-esteem increased trait anxiety values, acting serially through all three mediators (EA, EC, and MR) (β = 0.0013, *SE* = 0.00, 95% CI: -0.00, 0.00). Indirect effect 5 (Ind_5_ = *a*_2_*b*_2_) revealed that high self-esteem enhances EC levels, sequentially decreasing trait anxiety values. Indirect effect 6 (Ind_6_ = *a*_2_*d*_32_*b*_3_) indicated that higher self-esteem was associated with lower levels of trait anxiety acting serially through MR (β = -0.0634, *SE* = 0.00, 95% CI: 0.15, 0.45). Finally, indirect effect 7 (ind_7_ = *a*_3_*b*_3_), revealed that greater levels of self-esteem were associated with higher levels of MR what, in turn, decreased trait anxiety values (β = -0.1069, *SE* = 0.04, 95% CI: -0.20, -0.03). The analysis diagram of model B is shown in Figure 2.

To determine which of the indirect effects had more statistical weight, we performed contrast analyses between mediators. Table 3 shows the statistically significant contrasts with a CI of 95%. Considering the sign of the coefficients, the analyses showed that two of the indirect effects had higher statistical weigh: (1) The relationship of self-esteem on trait anxiety through EA and EC (Ind_2_ = *a*_1_*d*_21_*b_2_*) and, (2) The effect of self-esteem on trait anxiety through EA and MR (Ind_3_ = *a*_2_*d*_31_*b*_3_). Hence, these results indicated that, on the one hand, students with higher levels of self-esteem presented lower levels of EA and lower levels of EC what, in turn, increased trait anxiety values. On the other hand, participants with higher levels of self-esteem showed lower levels of EA and lower levels of MR what, in turn, increased trait anxiety values.

## Discussion

Literature up to date has been focused on the direct relationship between self-esteem, trait EI, and anxiety (Manna et al., 2016; Hartman et al., 2017; Mestre et al., 2017). However, little is known about the role of trait EI as a mediator of the relationship between self-esteem and anxiety. To the best of our knowledge, this is the first study that provides evidence about this mediation process differentiating between state and trait. Moreover, this research expands existing knowledge by exploring a model that considered trait EI (EA, EC, and MR) as mediator variable that enhances the effects of self-esteem on both types of anxiety among a sample of university students from the University of Cádiz.

With regard to the sample’s characteristics, the analyses showed that students displayed low levels of state and trait anxiety, relatively high self-esteem, and adequate levels of EA, EC, and MR for both men and women. Likewise, as the literature reflects, there are no statistically significant differences in the levels of anxiety, self-esteem, and trait EI dimensions according to the gender (Joseph and Newman, 2010; Tang and Sun, 2018). A positive and statistically significant correlation between age and self-esteem was found, indicating that the older the age of the students, the greater the self-esteem levels. However, as the hierarchical regression analysis showed, this relationship did not affect the effect of self-esteem and trait EI on state and trait anxiety.

Additionally, our results are consistent with Spielberger’s differentiation between anxiety as an emotional state (state anxiety) and anxiety as a personality trait (trait anxiety) (Spielberger, 1966, 1972, 1989). As Cattell and Scheier (1961) pointed out, the experience of an immediate emotional state, transient and modifiable in time, is not the same as having a more or less stable personality trait, determined by a tendency to perceive or not certain stimuli as threatening (Spielberger, 1972, 1989; Spielberger and Díaz-Guerrero, 1975). In this sense, and as confirmed by the positive and statistically significant correlations between state and trait anxiety obtained, individuals with higher levels of trait anxiety are more susceptible to stress, responding to several situations as if they were dangerous or threatening, showing state anxiety more frequently and with greater intensity than those with low trait anxiety (Spielberger et al., 1970).

Regarding the relationship between self-esteem and anxiety, we found common influence processes for state and trait anxiety. Our results confirmed that self-esteem scores explained and predicted both, state and trait anxiety values (Sowislo and Orth, 2013; Bajaj et al., 2016). This explanatory capacity is increased by 8% when accounting for trait EI subscales (EA, EC, and MR). However, the percentage of variance explained by self-esteem varied depending on the type of anxiety studied. Regarding state anxiety, the explanatory power of self-esteem was 13% and increased up to 21% when including trait EI dimensions. In relation to trait anxiety, the explanatory power of self-esteem in trait anxiety was 45% and increased up to 53% when introducing EA, EC, and MR.

Considering the direct relationships, we found differences depending on the trait EI dimensions and the type of anxiety involved. For state anxiety, the direct effects showed that a decrease in their levels is predicted through the increases in the levels of both, self-esteem and MR. Therefore, university students from our sample who rely on their own abilities to cope with threatening situations and who perceive themselves able to repair their emotional states experienced lower levels of state anxiety. These results are in line with Bigman et al. (2016) who found that positive expectations regarding emotion regulation lead to desired emotional outcomes. Regarding trait anxiety, the results of the direct effects revealed that a decrease in their levels is predicted, in addition to an increment of self-esteem and MR values, by an increase of EC and a decrease of EA. Hence, students in this research who trust in their own abilities to face hostile situations, who do not pay much attention to their emotions but who believe in their competencies to clearly perceive and repair their emotional states, showed lower levels of trait anxiety. On the contrary, sample undergraduates who pay much attention to their emotional states will present higher levels of trait anxiety. These findings, especially the results of trait anxiety, are in accordance with previous research revealing positive and direct relationships between EA and anxiety, and negative ones between EC and MR anxious responses (Aradilla-Herrero et al., 2014; Gómez-Díaz et al., 2017; Pena and Losada, 2017).

Regarding the statistically significant indirect effects found in our preliminary research, it is confirmed the negative relationship between self-esteem and anxiety found in the literature (Salovey et al., 2002; Cheung et al., 2015; Edo-Gual et al., 2015). In this line, we again find common elements in the process by which trait EI influence the role of self-esteem as a protective factor against state and trait anxiety. In both types of anxiety, the ability of self-esteem to reduce anxiety levels is enhanced by the effect that the former exerts on EC and EC on MR. Besides, high self-esteem is associated with a decrease in EA values, what in turn reduce trait anxiety levels. However, contrast analyses seem to revealed that the indirect effect of self-esteem on anxiety, acting serially through EA and MR, is the highest indirect effects for both, state and trait anxiety. Therefore, sample students with higher levels of self-esteem paid less attention to their emotions, which contributed to lower their MR levels, hence increasing state and trait anxiety values. With regard to trait anxiety, another indirect effect also stood out for its greater statistical weight and did not differ statistically from the previous one. Precisely, this indirect path is the relationship of self-esteem on trait anxiety through EA and EC. These results indicated that sample students with higher levels of self-esteem presented lower levels of EA and lower levels of EC what, in turn, increased trait anxiety values.

Given the positive relationship between all the three dimensions of trait EI evaluated (EA, EC, and MR), the negative association found between self-esteem and EA is a determinant element. The preliminary results of the mediation models showed that the effect of self-esteem on EA, and the influence that the latter had on EC and MR, wields an indirect mediated effect with the power to change the effect that self-esteem wields on both types of anxiety. In this sense, when higher levels of self-esteem are associated with lower EA, the protective role of self-esteem becomes a risk factor, given that ignoring current emotional states influence on how clearly sample subjects perceive and repair them, which leads to an increase in state and trait anxiety.

Our preliminary results partially support the Terror Management Theory (Greenberg et al., 1986) since we have found that self-esteem has a direct and mediated negative relationship with both state and trait anxiety. Students from our sample with high self-esteem may have more positive attitudes toward life. Therefore, anxiety buffers, such as self-esteem, allow undergraduates to cope with fears more easily (Hansen et al., 2010). However, sample individuals with higher levels of self-consciousness sometimes have a more negative life perspective than those with reduced self-awareness (Taubman-Ben-Ari and Noy, 2010). In this sense, self-esteem can operate in the opposite way.

Following the Theory of Cognitive Dissonance (Festinger, 1957) high levels of self-esteem can also predict lower EA values. According to this theory, two thoughts, relevant to the person, can be consonant if one follows the other naturally (e.g., if I trust my abilities, I feel satisfied with myself and with my competencies, and I will have lower anxiety levels). But these thoughts can also be dissonant if one comes into conflict with the other (e.g., if I want to trust my abilities, I need to know myself and my emotional states, but if I attend to my emotional states I will have more anxiety). The dissonant thoughts imply an uncomfortable psychological state that leads people to reduce this dissonance. In this sense, holding beliefs, even after they are proven to be defective, creates a cognitive dissonance regarding current information and past behavior, and the way to alleviate is simply rejecting new information. Hence, people will avoid attending to some information, such as their emotional states, and will focus on increasing their self-esteem to reduce anxiety levels. However, and as the data of this preliminary study showed, the desired effects are not reached, but the opposite. The sign of the influence of self-esteem on anxiety is reversed, turning it from a protection factor to a risk factor, due to the effect of EA on EC and MR, dimensions directly related to anxiety. In this respect, given our results, we consider that the most appropriate way to decrease anxiety levels for sample students (both state and trait anxiety) is to modify the idea that paying attention to our emotional states inevitably leads us to be aware of potential threats making us doubting about our abilities to face them. Hence, to modify our emotional states is necessary to clearly perceive our emotions and its causes (Lopes et al., 2012; Mestre et al., 2016, 2017).

The model generated to explore the mechanism by which trait EI (EA, EC, and MR) mediate the relationship between self-esteem and anxiety seems to be more elaborate for trait than for state anxiety and would encompass it. Therefore, the elements that act as protection and (or) risk factors for trait anxiety embrace the ones that act as protection and (or) risk factors for state anxiety. In this line of reasoning, the design of intervention programs aimed at enhancing the protective effect of self-esteem and trait EI on anxiety in university students from our sample should begin focusing on reducing trait anxiety, since this would result in a decrease of state anxiety. Therefore, we consider that strategies aimed at increasing self-esteem, EC, and MR should be incorporated early on. However, it is essential to include strategies to encourage sample students to pay adequate attention to their emotions. The opposite could reverse the protective ability of high self-esteem turning it into a risk factor, given that low levels of EA serially and directly affects EC and MR, which would increase both state and trait anxiety.

This research is not exempted from limitations, such as those associated with the use of self-report in the data collection besides the limitations of cross-sectional studies. Likewise, it will be necessary to increase the sample size and to test this model in other populations, in order to generalize our results.

However, despite the limitations, this study makes a considerable contribution exploring the mechanisms through which the feeling of satisfaction with oneself influences both the way of perceiving and responding to certain stimuli and potential threats and how this relationship is modified and explained better through the trait EI. Consequently, although self-esteem levels are a focus of interest for interventions, this study showed that including trait EI (EA, EC, and MR) increase the efficiency and profitability of these practical applications.

Finally, future research should focus on assessing the constructs studied using instruments based on ability models. Moreover, Monte Carlo simulation should be perform to determine the statistical power of indirect effects. It is important to note the need to investigate the levels of EA that prevent from the harmful effects that it may have on protective factors such as self-esteem, EC, and MR. In this sense, it is crucial to explore from which levels EA affects anxiety, as well as the levels from which EA affect the relationship between self-esteem, EC, and MR on both state and trait anxiety.

## Ethics Statement

All the procedures carried out in this study with human participants were developed in accordance with the ethical standards of the National Research Committee and with the Declaration of Helsinki of 1964 and its subsequent amendments or comparable ethical standards. Written informed consent was obtained from all individual participants included in the study. There is no conflict of interest linked to any of the authors associated with this study.

## Author Contributions

RG developed the study design, survey creation, performed the data collection and the data analysis, and contributed to writing the manuscript. RG-M contributed to data interpretation and writing the manuscript, and approved the final version of the manuscript. AZ contributed to project design and administration, manuscript revision. Finally, AM-C and PG-O contributed to the project design, data analysis, and writing the manuscript.

## Conflict of Interest Statement

The authors declare that the research was conducted in the absence of any commercial or financial relationships that could be construed as a potential conflict of interest.
